# Maternal Deprivation Increases Anxiety- and Depressive-Like Behaviors in an Age-Dependent Fashion and Reduces Neuropeptide Y Expression in the Amygdala and Hippocampus of Male and Female Young Adult Rats

**DOI:** 10.3389/fnbeh.2018.00159

**Published:** 2018-08-07

**Authors:** Alexandra S. Miragaia, Guilherme S. de Oliveira Wertheimer, Amanda C. Consoli, Rafael Cabbia, Beatriz M. Longo, Carlos E. N. Girardi, Deborah Suchecki

**Affiliations:** ^1^Departamento de Psicobiologia, Universidade Federal de São Paulo, São Paulo, Brazil; ^2^Department of Epidemiology, Graduate School of Public Health, University of Pittsburgh, Pittsburgh, PA, United States; ^3^Departamento de Fisiologia, Universidade Federal de São Paulo, São Paulo, Brazil

**Keywords:** maternal deprivation, emotional behavior, NPY, amygdala, hippocampus

## Abstract

Maternal deprivation for 24 h produces an immediate increase in basal and stress-induced corticosterone (CORT) secretion. Given the impact of elevated CORT levels on brain development, the goal of the present study was to characterize the effects of maternal deprivation at postnatal days 3 (DEP3) or 11 (DEP11) on emotional behavior and neuropeptide Y immunoreactivity (NPY-ir) in the basolateral amygdala (BLA) and dorsal hippocampus (dHPC) of male and female rats. Litters were distributed in control non-deprived (CTL), DEP3, or DEP11 groups. In Experiment 1, within each litter, one male and one female were submitted to one of the following tests: novelty suppressed feeding (NSF), sucrose negative contrast test (SNCT), and forced swimming test (FST), between postnatal days 52 and 60. In Experiment 2, two males and two females per litter were exposed to the elevated plus maze and 1 h later, perfused for investigation of NPY-ir, on PND 52. The results showed that DEP3 rats displayed greater anxiety-like behavior in the NSF and EPM, compared to CTL and DEP11 counterparts. In the SNCT, DEP3 and DEP11 males showed less suppression of the lower sucrose concentration intake, whereas all females suppressed less than males. Both manipulated groups displayed more immobility in the FST, although this effect was greater in DEP3 than in DEP11 rats. NPY-ir was reduced in DEP3 and DEP11 males and females in the BLA, whereas in the dHPC, DEP3 males showed less NPY-ir than DEP11, which, in turn, presented less NPY-ir than CTL rats. Females showed less NPY-ir than males in both structures. Because the deprivation effects were more intense in DEP3 than in DEP11, in Experiment 3, the frequency of nursing posture, licking-grooming, and interaction with pups was assessed upon litter reunion with mothers. Mothers of DEP11 litters engaged more in anogenital licking than mothers of DEP3 litters. The present results indicate that maternal deprivation changed affective behavior with greater impact in the earlier age and reduced the expression of NPY in emotion-related brain areas. The age-dependent differential effects of deprivation on maternal behavior could, at least in part, explain the outcomes in young adult rats.

## Introduction

Affective disorders are highly prevalent with profound impact on society (Baxter et al., 2013). Depression, for instance, has been recognized by the World Health Organization as the leading cause of disability worldwide, and it is estimated that 300 million people of all ages suffer from depression (WHO, 2018). These disorders result from the interaction between genetic background and environmental adversity during critical periods of development, such as infancy and adolescence (Plotsky et al., 1998; Caspi et al., 2003). Epidemiological and experimental studies have shown that loss of parental care, due to death of one or both parents, divorce or abandonment, are major risk factors for the development of mental disorders and dysregulation of the hypothalamic-pituitary-adrenal (HPA) axis activity in adolescence and adulthood (Agid et al., 1999; Tyrka et al., 2008; Tofoli et al., 2011). Particularly, a recent epidemiological study showed that loss of the mother by accidents and homicide has greater impact on young men than women and that parental death at an earlier (0- to 5-years-old), rather than later age (6- to 17-years-old) confers higher risk for depression (Berg et al., 2016). In a large longitudinal study with Canadian children, loss of a parent, either by death, divorce, or separation, between 4 and 8 years of age is a strong predictor of depression at 16–20 years of age, for girls but not boys (Bellamy and Hardy, 2015), indicating a sex-dependent impact of early parental loss.

Maternal deprivation for 24 h in rats is a paradigm used to mimic the transient loss of maternal care during the stress hyporresponsive period (SHRP) and represents the removal of inhibitory regulation that the mother exerts on the offspring’s HPA axis (Hofer, 1994). The SHRP is an extremely important developmental phase, lasting from postnatal day (PND) 4 to PND 14, and characterized by adrenal insensitivity to its trophic hormone, ACTH (Witek-Janusek, 1988) and to most stressors, ensuring the maintenance of low and stable levels of corticosterone (CORT), a requirement for proper brain development (for review, see Rosenfeld et al., 1992). Maternal behaviors such as licking/grooming and nursing are responsible for inhibiting the secretion of ACTH and CORT, respectively (Cirulli et al., 1992; Rosenfeld et al., 1993; Suchecki et al., 1993; van Oers et al., 1998). In a seminal study, Levine et al. (1991) demonstrated that the immediate effects of maternal deprivation on basal, stress-, and ACTH-induced CORT secretion were dependent on the pups’ age. Thus, at PND 3, i.e., before the onset of the SHRP, 24 h of deprivation results in a slight increase in basal and stress-, but not in ACTH-induced CORT levels, whereas at PND 11, e.g., during the SHRP, it leads to a very robust CORT response to all challenges (Levine et al., 1991).

The long-term effects of maternal deprivation appear to depend on the age when it takes place as well as when the animals are tested. For instance, maternal deprivation on PND 3 reduces hippocampal neurogenesis in prepubertal females, but not in males (Oomen et al., 2009); it also impairs long-term potentiation and hippocampal-dependent learning of adult males under unstressed conditions, but improves these functions when animals are tested under high CORT levels or stressful conditions (Oomen et al., 2010), suggesting that this early life stress may prepare the animal to face adversity in adulthood in a more adaptive way (Loi et al., 2014). Nonetheless, adult male and female rats deprived on PND 3 display more anxiety-like behavior in the light-dark box (Faturi et al., 2010). When pups are maternally deprived on PND 9, male adolescent rats displayed increased anxiety-like behavior and social evasion in the social investigation test (Girardi et al., 2014), while adult males exhibit behavioral changes reminiscent of schizophrenia symptoms (Ellenbroek et al., 1998; Husum et al., 2002), and augmented striatal and cortical dopaminergic tonus and dopamine and serotonin concentrations in the amygdala (Rentesi et al., 2013). When imposed on PND 11, maternal deprivation results in anxiety-like profile in adult male and female rats (Faturi et al., 2010; Barbosa Neto et al., 2012) and reduces hippocampal levels of two inhibitory amino acid neurotransmitters in a sex-dependent way, taurine in males and GABA in females (Barbosa Neto et al., 2012). Interestingly, juvenile male and female rats deprived on PND 11 and tested in the open-field exhibit less anxiety-like behavior than control counterparts, spending more time in the central part of the apparatus (Suchecki et al., 2000).

In children, the cortisol response to medical examinations and vaccinations decrease from 6 to 12 months, while from 12 to 18 months of age, there is virtually no cortisol response to these events, indicating that the SHRP may also occur in humans. Interestingly, the suppression of cortisol response is moderated by the quality of parental care, i.e., children of low-sensitive parents and insecure attachment show higher cortisol stress response during this period (for review, see Gunnar and Cheatham, 2003). Studies designed to investigate the long-term impact of parental loss around the SHRP in humans are scarce and have resorted on post-institutionalized adolescents. Assessment of adopted adolescents (average age 12.9 years) with a history of orphanage-dwelling of approximately 2 years shows that they exhibit less risk-taking behavior and more depressive symptoms than adolescents raised by their biological parents (Loman et al., 2014). Regarding brain structural changes in early- or late-adopted (before or after 12-months-olds, respectively) 13- to 14-years-old adolescents, data show reduction of prefrontal cortex thickness and hippocampal volume, with greater impact on the late-adopted subgroup (Hodel et al., 2015). Moreover, late (after 15 months of age), but not early adopted 8- to 10-years-old children show greater amygdala activation, anxiety levels, and impaired emotional regulation (Tottenham et al., 2010). Collectively, these findings indicate that the 24 h maternal deprivation paradigm may be a valuable translational model for the study of short- and long-term consequences of neonatal adversity-induced vulnerability to psychiatric disorders.

Recently, the role of neuropeptide Y (NPY) in stress resilience has gained much attention. The evidence shows that NPY deficiency is involved in the pathophysiology of emotional and mood disorders (for review, see Heilig, 2004; Wu et al., 2011; Kormos and Gaszner, 2013; Thorsell and Mathé, 2017). Clinical data demonstrate a reduction of NPY cerebrospinal fluid levels of treatment-resistant depressive (Heilig et al., 2004) and posttraumatic stress disorder (PTSD) patients (Sah et al., 2009, 2014), whereas pre-clinical studies report reduced NPY levels in an animal model of PTSD (Cohen et al., 2012) and a potential therapeutic effect of this peptide in a model of PTSD, since intra-nasal administration of NPY in rats either prevents (Serova et al., 2013) or reverses (Serova et al., 2014) the behavioral alterations, suggesting that this neuropeptide could confer protection against stress-related psychopathologies.

Disruption of the mother-infant relationship by means of 3 or 6 h of maternal separation leads to increased anxiety- and depressive-like behaviors (Huot et al., 2001) and reduces NPY-immunoreactivity (ir; Husum and Mathé, 2002) and hippocampal levels (Jiménez-Vasquez et al., 2001). Data on the effects of maternal deprivation on NPY are scarce, but the evidence indicates an overall reduction: DEP9 animals exhibit lower NPY levels in the hippocampus and occipital cortex than control rats (Husum et al., 2002), while DEP3 and DEP11 male and female adolescents display reduced food intake during the rats’ active period and lower NPY-ir in the arcuate nucleus of the hypothalamus than controls (Wertheimer et al., 2016). Therefore, it appears as though neonatal stress leads to reduction of NPY in several brain areas involved with motivated and emotional behaviors, and this effect could represent a neurobiological underpinning of vulnerability to later psychopathology.

Based on the abovementioned evidence, we hypothesized that maternally deprived rats would display more anxiety and/or depressive-like behaviors in a series of behavioral tests and express less NPY in the basolateral amygdala (BLA), ventral hippocampus (vHPC) and dorsal hippocampus (dHPC), two emotional behavior-related brain areas.

## Materials and Methods

These studies only began after approval of the Ethics Research Committee of Universidade Federal de São Paulo, in accordance to the Guidelines of the Brazilian National Council for Control of Animal Experimentation (Protocols 187322 and 0340/12) and the National Institutes of Health guide for the care and use of Laboratory animals. All efforts were made to minimize the animals’ discomfort and the sample size.

### Breeding and Husbandry

Male and female 3-months-old Wistar rats [from Centro de Desenvolvimento de Modelos Experimentais (CEDEME) of Universidade Federal de São Paulo, São Paulo, Brazil] were mated in the Department of Psychobiology, under controlled temperature (21 ± 3°C) and lighting (12 h light/dark cycle; lights on at 7:00 h). Briefly, one male was housed with two virgin females for 10 days. Females were individually housed 18 days after the beginning of mating and wood shavings and paper towel were provided as nesting material. Cages were inspected daily and date of birth was designated PND 0. On PND1, litters were adjusted to four females and four males and randomly assigned to one of three groups: control, non-deprived (CTL), maternal deprivation on PND 3 (DEP3) or on PND 11 (DEP11). Cages were cleaned once every 3 days, by replacing half of the bedding with clean material, and providing more nesting material. Unless stated otherwise, animals had free access to food and water during the entire study.

### Maternal Deprivation

Pups assigned to DEP3 or DEP11 groups were removed from the presence of the dam at 10:00 h and placed in cages containing bedding from the maternity cage. There they remained undisturbed for 24 h on a heating pad set at 30–33°C. After reunion with the mother, pups were not disturbed until weaning, which happened on PND 21. At that time, pups were separated by sex and housed as a litter (siblings were kept together, four animals per cage; Experiment 1) or in pairs (Experiment 2) with food and water provided *ad libitum*, under the same environmental conditions described above.

### Experiment 1

#### Animals

A total of 30 litters, distributed in three groups (10 litters per group) were used in this experiment. Testing was carried out between PND 52 and PND 60, the equivalent to the transition period from late adolescence to early adulthood. This age range was chosen because in humans, this period seems to mark the onset of manifestations of emotional problems (Patten et al., 2006) and females were tested during the estrous phase; from PND 52 on, vaginal smears were collected and the female was tested once estrous phase was detected. This phase of the cycle was chosen because, together with proestrous, females display less anxiety-like behavior in classical tests (Marcondes et al., 2001; Ramos-Ortolaza et al., 2017) and in diestrous, females show more depressive-like behavior (Walf and Frye, 2010). Moreover, during diestrous and metestrous, when estradiol levels are low, females behave more similarly to males (ter Horst et al., 2012), hindering possible sex differences. To avoid litter effect, one male and one female from each litter were submitted to one of three behavioral tests; the remaining couple of animals were decapitated under basal conditions.

#### Body and Adrenal Relative Weights

One male and one female were taken from each litter and weighed. They were decapitated and the adrenals, removed, cleaned of surrounding fat, and weighed immediately. Relative adrenal weight was calculated according to the following equation: (sum of both adrenals weight [mg]/body weight [g]) × 1000).

#### Behavioral Assessment

##### Novelty suppressed feeding (NSF)

This test is used to assess anxiety-like behavior in rodents and was validated by (Bodnoff et al., 1988). Fifty-nine adolescent rats were food deprived for 48 h before the test: CTL (10 females and 10 males), DEP3 (10 females and 9 males), and DEP11 (10 females and 10 males). At the end of the fasting period, they were transferred to the testing room and habituated for 60 min. Each rat was placed in the corner of an unknown plastic cage (110 cm long × 60 cm wide × 40 cm walls), where a previously weighed pellet of chow was placed in the other extremity of the cage. The test was video-recorded and lasted 10 min, during which the animal could explore the new environment. The latency for the first bite and the total amount of chow eaten were recorded and, after the testing period, the rats were returned to their home-cages, where food pellets were available to be consumed for 30 min. The post-test food intake was carried out to eliminate the possibility of lack of motivation to eat, which would be detected in the known environment. After each test, the plastic cage was cleaned with alcohol 70%, to eliminate olfactory signs.

##### Sucrose negative contrast test (SNCT; Matthews et al., 1996)

This is a test used to assess motivational behavior of rats to engage in pleasurable activity. It is based on the consummatory response to a sudden decrease in concentration of a palatable sucrose solution, in which the suppression of a lesser palatable solution is expected. Sixty-one adolescent rats were used: CTL (10 males and 11 females), DEP3 (10 males and 10 females), and DEP11 (10 males and 10 females). The test was carried out for 3 consecutive days, and the rats were housed individually in home-cages during the evaluation period. In the first 2 days, two identical bottles were offered to the animals, one containing a 15% sucrose solution and the other, water. The bottles were switched between days to avoid any possible place preference. On the third day, the sucrose solution was changed to 2.1% (less palatable, hence, less attractive). Liquid intake (from each bottle) was measured every 24 h, by weighing the bottles, and sucrose preference index (SPI) was estimated by the equation: volume of sucrose intake/total liquid intake (sucrose + water). Reduced intake of 2.1%, compared to that of 15% sucrose solution is the expected behavioral outcome for rats displaying a hedonic profile (Verma et al., 2010; Girardi et al., 2014). The variation of SPI between days 2 (15%) and 3 (2.1%) was calculated as a measurement of suppressed intake of the less concentrated solution.

##### Forced swimming test (FST)

This classical test for evaluation of antidepressant drugs is based on learned helplessness from an inescapable situation (Porsolt et al., 1977). The protocol used in the present study was modified by Detke et al. (1995) and Lucki (1997). Sixty animals were used, corresponding to 10 animals/group/sex. On the first day, each rat was trained individually, for 15 min, in an acrylic transparent cylinder, measuring 20 cm in diameter and 50 cm in height and containing water up to 30 cm (24 ± 1°C). Twenty-four hours later, the rats were placed, individually, in the cylinder for 5 min (testing session) and the sessions were filmed by a camera positioned in front of the cylinder for posterior behavioral analysis; frequency of the predominant behavior at every 5 s was recorded, including swimming (movements throughout the swim cylinder), climbing (upward directed movements of the forepaws along the cylinder walls), and immobility (when the rat stopped all active behaviors and remained floating in the water with minimal movements, with its head just above the water). After each training and testing session, the animals were dried with a soft towel and placed back in their home-cages, whereas the cylinders were washed to eliminate olfactory clues.

### Experiment 2

#### Animals

Twenty-eight litters were distributed in CTL (nine litters), DEP3 (10 litters), and DEP11 (nine litters). In each litter, two males and two females were tested in the elevated plus maze, whereas the remainder of the litter was not tested and provided baseline values of parameters published previously (Wertheimer et al., 2016) and NPY-ir.

#### Elevated Plus Maze (EPM)

On PND 52, 62 animals (CTL group: 11 males and 9 females; DEP3 group: 10 males and 12 females; DEP11 group: 10 males and 10 females), females tested during estrous (detected as explained above), were exposed to the EPM for assessment of anxiety-like behavior. The test was conducted between 8:00 and 12:00 h in a maze constructed of wood, painted in brown, with four arms forming a cross 50 cm above the ground. All arms were 50 cm in length and 10 cm wide and two opposite arms, denominated closed, had 40 cm enclosing walls, while the open arms contained a small 0.5 cm ledge, which served as a tactile guide for the animals (Cohen et al., 2003). The test was 5 min long, and each rat was brought to the EPM cubicle and placed in the central square of the apparatus with its nostril facing one of the open arms (Pellow et al., 1985), under a 12.5 lux, red light. Each session was video-recorded for posterior behavioral analysis. After each test, the EPM was wiped out with a 20% alcohol solution.

The parameters considered for analysis included the behaviors reflecting the exploratory activity of the aversive arms: (1) percentage of visits into the open arms (%VOA – represented by the ratio between entries into the open arms and total entries in all arms); (2) percentage of time in the open arms (% TOA – time spent in the open arms/300 = time of the test); (3) squares crossed in the OA; and (4) frequency of visits to the extremity of the OA (number of times that the rat reached the extreme edge of these arms). Entries were considered when the rats placed all four paws inside the arms and the same was true for motor activity.

Anxiety index was calculated according to Mazor et al. (2009), considering the frequency and time spent in the open arms in relation to the total exploration of the apparatus. Anxiety index stands between 0 and 100 and higher indices indicate higher anxiety level.

$$Anxiety\;index\;(\%)=1-\left[\frac{\left(\frac{Time\;in\;OAs}{Total\;time\;of\;test}\right)+\left(\frac{Entries\;in\;the\;OAs}{Total\;entries}\right)}{2}\right]\times 100$$

#### Tissue Preparation

After the EPM, each animal was taken back to its home-cage, together with its pair, and 15 min later, one to two males and females from each litter were anesthetized with sodium thiopental and submitted to gravity perfusion, only when they did not display any pain response, using the AutoMate In Vivo Perfusion System #11-800. The perfusion needle was inserted into the left ventricle and positioned in the ascendant aorta, the descendent aorta was clamped above the liver, and the right auricle was sectioned. After these procedures, 200 mL of heparinized saline solution at 0.9% was inserted into the left ventricle. Following the saline wash, the tissue was fixed using 250 mL of PFA at 4% in a phosphate buffered saline solution (PBS, 0.1 M, pH 7.4). At the end of perfusion, animals were decapitated and their heads kept at 4°C in a solution of PFA at 4% in 0.1 M PBS. The following day, brains were removed from the skulls and infiltrated gradually with sucrose up to 30% in 0.1 M PBS for crioprotection. After 48 h in the sucrose infiltration, brains were frozen on containers made of aluminum foil floating in a solution of dry ice and methanol.

#### Immunohistochemistry

Brains were cut into 50 μm coronal sections that spanned from bregma 2.3 to 3.3 mm for the BLA and bregma 4.3–4.6 mm for the dHPC, using a Leica CM1850 cryostat at −20°C, according to the stereotaxic coordinates of the rat brain atlas (Paxinos and Watson, 1998). Sections were sequentially placed in the six wells filled with PBS and sucrose anti-freezing solution of a 24-multiwell plate.

Sections stored in PBS/sucrose anti-freezing solution at −20°C were washed extensively with PBS and then incubated at 4°C for 48 h with anti-NPY developed in rabbit (Sigma-Aldrich, Brazil). For the analysis of BLA, vHPC, and dHPC, a 1:1000 dilution was used. After being washed in PBS, all sections were incubated at room temperature for 2 h using a biotinylated secondary anti-rabbit antibody (Vector Laboratories, Burlingame, CA, United States) in a 1:600 dilution. Immunocomplex was made visible by using the Vectastain Elite ABC kit (Vector Laboratories, Burlingame, CA, United States) according to manufacturer’s instructions and then stained with DAB (0.5 mg/mL) for 15 min. Finally, sections were mounted onto gelatine-coated microscope slides and allowed to air dry. Slides were then covered with mounting media and coverslips.

The images were visualized using an Olympus BX50 (Olympus, Japan) light microscope and recorded digitally. Neurons marked on both hemispheres of the BLA and of the vHPC and dHPC were individually counted by A.S.M., who had no knowledge of the experimental group or sex of each animal and NPY immunoreactivity (NPY-ir) in these structures was estimated by averaging cell counts of 6 sections per structure.

### Experiment 3

Previous studies demonstrate that aspects of maternal care can modulate the activity of the pup’s HPA axis activity; thus, replacement of nursing and anogenital licking during the maternal deprivation period prevents, almost completely, the effects of maternal absence at all levels of the HPA axis (Rosenfeld et al., 1993; Suchecki et al., 1993; van Oers et al., 1998). Moreover, natural variations in maternal care result in distinct behavioral profiles in adulthood, inasmuch as the offspring of mothers engaging in high levels of arched-back nursing and anogenital licking display low levels of fear and stress reactivity (Caldji et al., 1998; Champagne et al., 2003). Therefore, we recorded the frequency of different nursing postures: passive, when the mother laid on its back and pups approached the nipples; blanket, when the mother laid on top of the litters; arched-back nursing, when the mother arched its back to allow pups to reach its nipples (Rees et al., 2005); licking-grooming, the mother licked the pup’s body; and anogenital licking-grooming, when the mother licked the anogenital area of the pup.

#### Animals

Twelve litters were used, seven DEP3 and five DEP11. At the end of the deprivation period, litter and mother were reunited and maternal behavior was recorded during 1 h, at 10:00, 14:00, and 17:30 h. Every 3 min, maternal behavior (s) was (were) recorded, making up for 20 recordings per hour at each time-point; the sum of all recordings provided the total frequency. Importantly, the behaviors were not mutually exclusive and the mother could engage in two behaviors at the same time; in that case, both were recorded.

### Statistical Analysis

Physiological (body weight and relative adrenal weight), behavioral parameters from novelty suppressed feeding (NSF) and forced swimming test (FST), EPM, and NPY-ir were analyzed by a two-way ANOVA with sex (male, female) and group (CTL, DEP3, DEP11) as main factors. Data on daily SPI in the sucrose negative contrast test (SNCT) were analyzed by a three-way ANOVA for repeated measures with sex, group, and day as main factors. The difference of SPI between days 3 (2.1%) and 2 (15%) was analyzed by a two-way ANOVA with sex and group as main factors. *Post hoc* analyses were done by the Newman–Keuls test and the level of significance was established at *p* < 0.05.

Analysis of maternal behavior was done by the Mann–Whitney *U*-test, with the level of significance set at *p* < 0.05.

Cohen’s *d* effect size was calculated for all behavioral and neurochemical data. This index measures the magnitude of an effect (or percentage of overlap between groups) based on the means and standard deviations of each comparison group; the calculator is available at https://www.uccs.edu/lbecker/. Interpretation of the *d* values can be found at https://www.uccs.edu/lbecker/effect-size, where values between 0.5 and 0.8 represent moderate effect, whereas those above 0.81 represent large effect.

## Results

### Experiment 1

#### Body and Relative Adrenal Weights (**Table 1**)

Main effects of sex [*F*_(1,56)_ = 26.058; *p* < 0.00001] and group [*F*_(2,56)_ = 4.731; *p* < 0.02] were revealed for body weight. Females were lighter than males (*p* < 0.0002) and DEP3 and DEP11 rats, regardless of sex, were lighter than their CTL counterparts (*p* < 0.04, for both groups).

**Table 1:** Body and relative adrenal weights of adolescent male and female rats, kept with the mother – control (CTL) or maternally deprived on postnatal days 3 (DEP3) or 11 (DEP11).

	Body weight (g)		
	CTL	DEP3	DEP11
Males	232.64 ± 29.9 (11)	201.10 ± 32.9 (10)^∗^	212.00 ± 20.9 (10)^∗^
Females	192.09 ± 19.8 (11)¥	179.90 ± 17.4 (10)^∗^¥	179.90 ± 18.8 (10)^∗^¥

	**Relative adrenal weight**		
	**CTL**	**DEP3**	**DEP11**

Males	0.19 ± 0.02	0.23 ± 0.05^∗^	0.21 ± 0.04
Females	0.26 ± 0.03¥	0.30 ± 0.02^∗^¥	0.29 ± 0.04¥

*Animals were not tested and the results are presented as mean ± SEM of the number of animals shown in parenthesis. ^∗^ – Different from CTL group; ¥ – different from males.*

Regarding the relative adrenal weight, there were also main effects of sex [*F*_(1,56)_ = 69.111; *p* < 0.00001] and group [*F*_(2,56)_ = 5.451; *p* < 0.007]. Females’ adrenals were heavier than males’ and those of DEP3 were heavier than adrenals of CTL rats (*p* < 0.006); DEP11 males and females tended to have heavier adrenals than CTL counterparts (*p* = 0.07; **Table 1**), with, respectively, moderate and high magnitudes for males and females (**Table 4**).

#### Novelty Suppressed Feeding (**Figure 1**)

Two-way ANOVA detected main effect of group for the latency to eat the pellet [*F*_(2,54)_ = 5.925; *p* < 0.005]; both DEP3 males and females took longer to eat than CTL and DEP11 (*p* < 0.05). As for the amount consumed, females ate less than males [*F*_(1,54)_ = 30.682; *p* < 0.0001].

**FIGURE 1:**
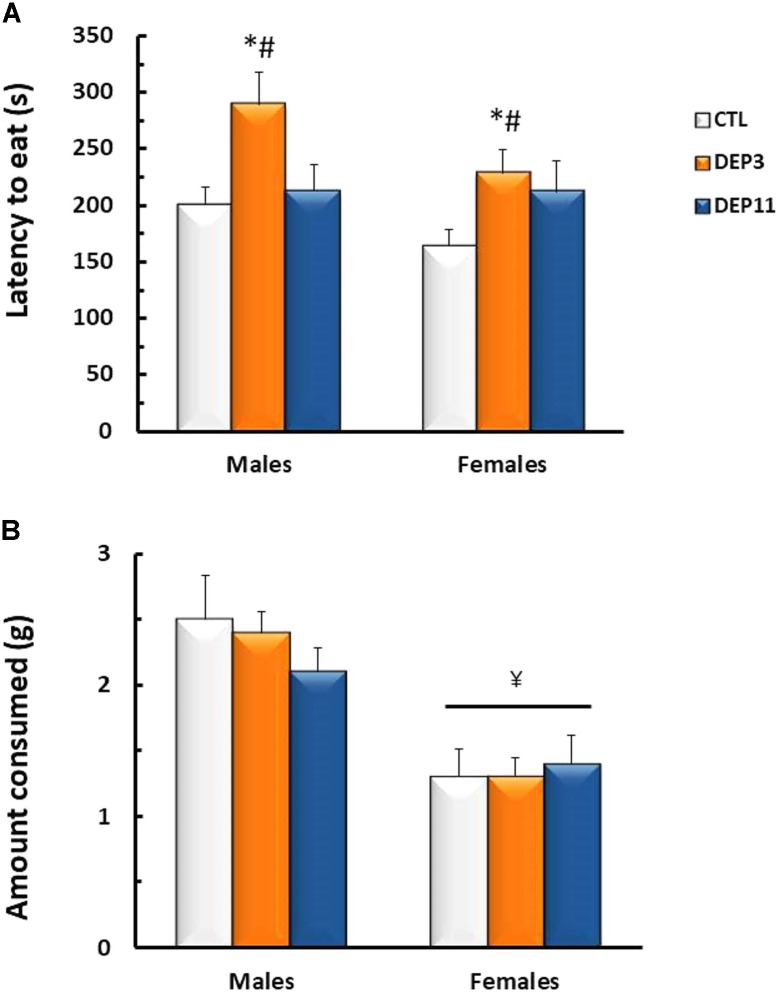
Latency to the first bite in the chow pellet **(A)** and amount of chow consumed **(B)** in a 10 min session of novelty suppressed feeding test. Animals were maintained with their mothers (CTL), deprived on postnatal day (PND) 3 (DEP3) or on PND 11 (DEP11) and tested between PNDs 52 and 60. Data are presented as mean ± SEM of 9–10 animals/group/sex. ^∗^ – Different from CTL group; # – different from DEP11 group; ¥ – different from respective male group.

#### Sucrose Negative Contrast Test (**Figure 2**)

Interactions between sex and day [*F*_(2,110)_ = 5.45; *p* < 0.01] and between group and day [*F*_(4,110)_ = 2.71; *p* < 0.04] were observed for sucrose preference. DEP3 and DEP11 males suppressed the intake of 2.1% sucrose solution less than CTL rats (*p* < 0.004), whereas all females, regardless of group, displayed less reduction of this solution intake than males (*p* < 0.0001).

**FIGURE 2:**
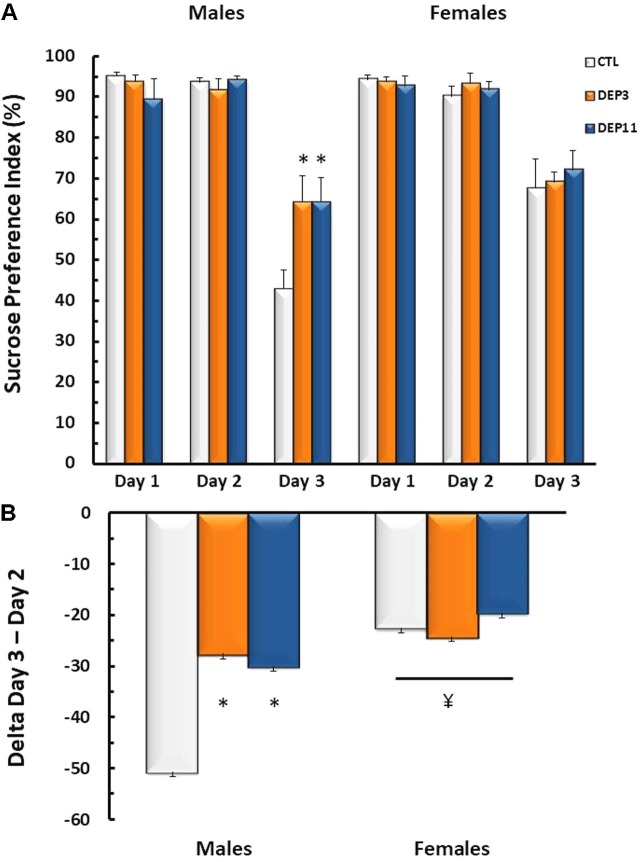
**(A)** Sucrose preference index and **(B)** variation of sucrose intake between Days 3 (2.1% sucrose concentration) and 2 (15% sucrose concentration) in the sucrose negative contrast test. Animals were maintained with their mothers (CTL), deprived on postnatal day (PND) 3 (DEP3) or on PND 11 (DEP11) and tested between PNDs 52 and 60. Data are presented as mean ± SEM of 10–11 animals/group/sex. ^∗^ – Different from CTL group; ¥ – different from respective male group.

#### Forced Swimming Test (**Figure 3**)

**FIGURE 3:**
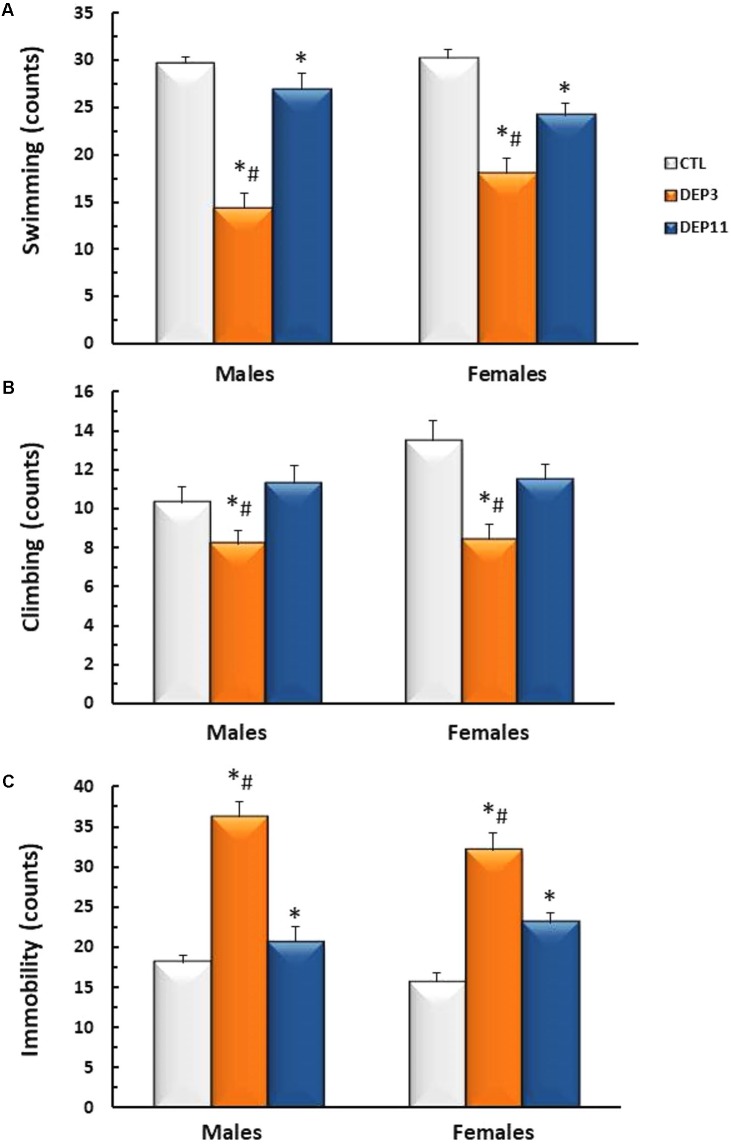
Frequency of swimming **(A)**, climbing **(B)**, and immobility **(C)** during a 5 min session of the forced swimming test. Animals were maintained with their mothers (CTL), deprived on postnatal day (PND) 3 (DEP3) or on PND 11 (DEP11) and tested between PNDs 52 and 60. Data are presented as mean ± SEM of 10 animals/group/sex. ^∗^ – Different from CTL group; # – different from DEP11.

Both DEP3 males and females swam less than DEP11 counterparts (*p* < 0.0006 for both comparisons), which, in turn, swam less than the respective CTL groups (*p* < 0.0005) (Group effect [*F*_(2,54)_ = 50.808; *p* < 0.0001]). Regarding the frequency of climbing behavior (**Figure 3B**), group effect was detected [*F*_(2,54)_ = 10.5; *p* < 0.0005] and DEP3 males and females displayed less climbing than both DEP11 and CTL counterparts (*p* < 0.001, for both). Frequency of immobility (**Figure 3C**) was higher in DEP3 than DEP11 rats (*p* < 0.0005), which in turn, was higher than CTL counterparts (*p* < 0.005) [*F*_(2,54)_ = 62.53; *p* < 0.0001].

### Experiment 2

#### Elevated Plus Maze

##### Percentage of entries in the OA (**Figure 4A**)

An interaction between sex and group was detected [*F*_(2,56)_ = 3.808; *p* < 0.03] and, although there were no differences among males, DEP3 females entered the open arms significantly less than both CTL and DEP11 counterparts (*p* < 0.005 for both comparisons).

**FIGURE 4:**
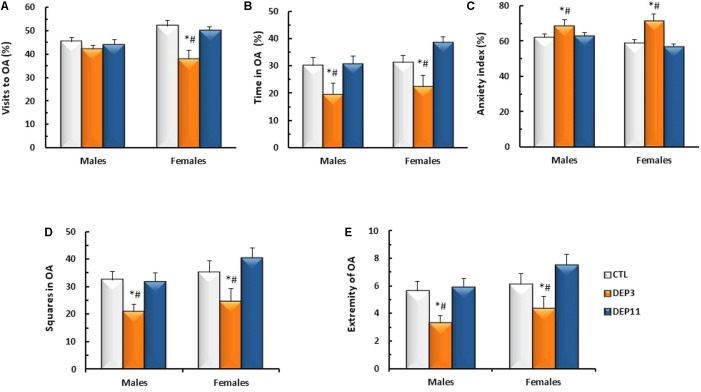
Percentage of visits **(A)** and time spent **(B)** in the open arms (OA); anxiety index **(C)**; number of squares crossed in the OA **(D)** and number of times that the animals reached the far end of the OA **(E)** during a 5 min session in the elevated plus maze. Animals were maintained with their mothers (CTL), deprived on postnatal day (PND) 3 (DEP3) or on PND 11 (DEP11) and tested between PNDs 52 and 60. Data are presented as mean ± SEM of 9–12 animals/group/sex. ^∗^ – Different from CTL group; # – different from DEP11.

##### Percentage of time in OA (**Figure 4B**)

Main effect of group was observed [*F*_(2,56)_ = 9.642; *p* < 0.0005], with both DEP3 males and females spending less time in the OA than CTL and DEP11 counterparts (*p* < 0.005 for both comparisons).

##### Anxiety index (**Figure 4C**)

Main effect of group was observed [*F*_(2,56)_ = 10.734; *p* < 0.0002], confirming that DEP3 rats, regardless of sex, displayed higher anxiety index than their CTL and DEP11 counterparts (*p* < 0.001 for both comparisons).

##### Squares crossed in the OA (**Figure 4D**)

Exploratory activity in the open arm was reduced in both male and female DEP3 rats, compared with the other groups (main effect of Group [*F*_(2,56)_ = 7.734, *p* < 0.001]).

##### Visits to the extremity of the OA (**Figure 4E**)

Again DEP3 males and females approached the extreme portion of the open arm less than CTL and DEP11 counterparts (main effect of Group [*F*_(2,56)_ = 8.193; p < 0.0008]).

#### Immunohistochemistry for NPY

An initial analysis considering condition (basal or post-EPM) did not reveal differences in NPY-ir for any of the regions evaluated [BLA: *F*_(1,60)_ = 0.000; *p* < 0.99; dHPC: *F*_(1,59)_ = 0.453; *p* < 0.50; vHPC: *F*_(1,59)_ = 1.494; *p* < 0.23]. Therefore, data were pooled across this variable.

##### Basolateral amygdala

**FIGURE 5:**
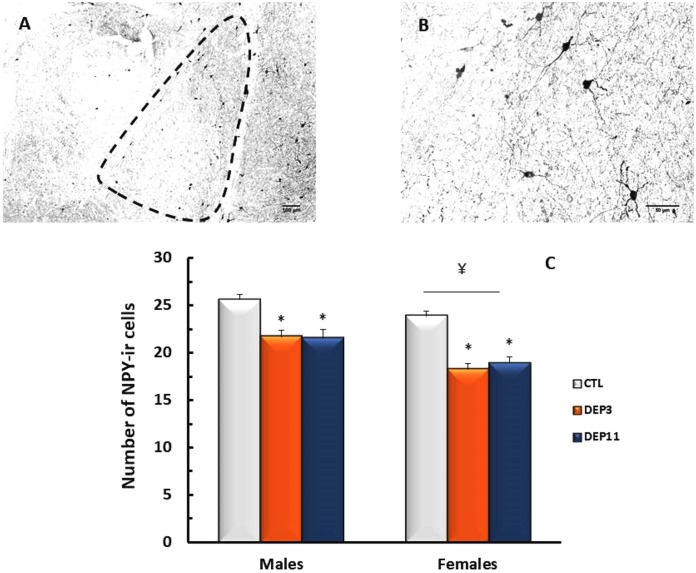
Photomicrograph of the basolateral amygdala (BLA) of one control rat at 10x **(A)** and 40x magnification **(B)**. Number of NPY-immunoreactive neurons in the BLA **(C)** of animals maintained with their mothers (CTL), deprived on postnatal day (PND) 3 (DEP3) or on PND 11 (DEP11) and perfused between PNDs 52 and 60. Data are presented as mean ± SEM of 11–15 males and 10–12 females/group. Scale bar in **A** = 100 μm; scale bar in **B** = 50 μm. ^∗^ – Different from CTL group; ¥ – different from respective male group.

NPY-ir in the BLA was reduced in both deprived groups compared to CTL (group effect [*F*_(2,66)_ = 27.71; *p* < 0.0001]); in addition, deprived females exhibited less NPY-ir than their male counterparts (*p* < 0.01 for DEP3 and *p* < 0.02 for DEP11; sex effect [*F*_(1,66)_ = 20.435; *p* < 0.0001]). These results are shown in **Figure 5**.

##### Ventral hippocampus

Main effect of sex [*F*(1,65) = 11.814; *p* < 0.001], but not of group or interaction between factors, was detected; females showed significantly less NPY-ir in the ventral hippocampus than males (*p* < 0.001). Values of NPY-ir in this area are shown in **Table 2**.

**Table 2:** Number of positive NPY-immunoreactive cells in the ventral hippocampus (vHPC) of adolescent male and female rats, kept with the mother – control (CTL) or maternally deprived on postnatal days 3 (DEP3) or 11 (DEP11).

	NPY positive neurons in the vHPC		
	CTL	DEP3	DEP11
Males	83.77 ± 15.4 (13)	85.54 ± 22.6 (11)	79.20 ± 16.6 (15)
Females	76.54 ± 9.2 (11)¥	60.70 ± 12.4 (10)¥	72.54 ± 13.8 (11)¥

*The results are presented as mean ± SD of the number of animals shown in parenthesis. ¥ – Different from males.*

##### Dorsal hippocampus

**FIGURE 6:**
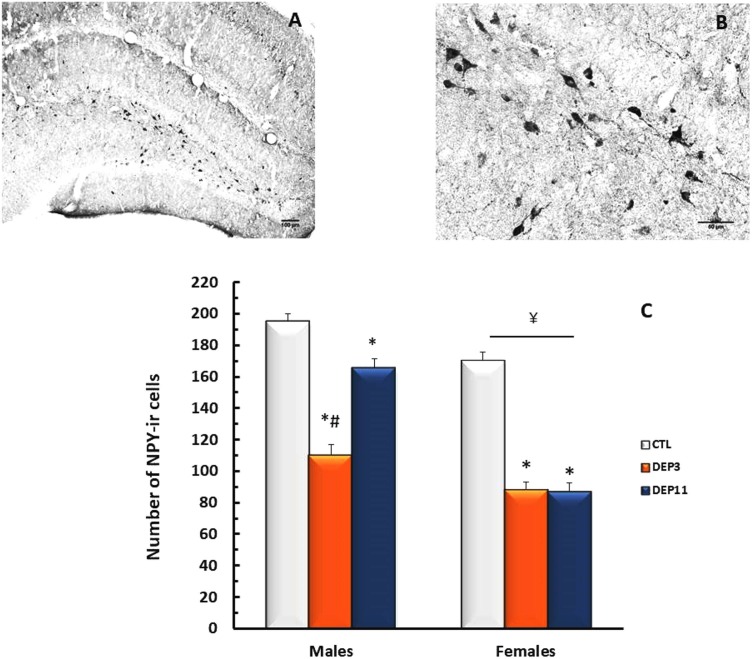
Photomicrograph of the dorsal hippocampus (dHPC) of one control rat at 10x **(A)** and 40x magnification **(B)**. Number of NPY-immunoreactive neurons in the dHPC **(C)** of animals maintained with their mothers (CTL), deprived on postnatal day (PND) 3 (DEP3) or on PND 11 (DEP11) and perfused between PNDs 52 and 60. Data are presented as mean ± SEM of 11–15 males and 10–11 females/group. Scale bar in **A** = 100 μm; scale bar in **B** = 50 μm. ^∗^ – Different from CTL group; # – different from DEP11; ¥ – different from respective male group.

An interaction between group and sex was shown [*F*_(2,65)_ = 17.403; *p* < 0.0001]. NPY-ir was lower in DEP3 than in DEP11 males (*p* < 0.001), which, in turn, was lower than in CTL counterparts (*p* < 0.001). In females, both manipulated groups displayed less NPY-ir than CTL group (*p* < 0.01 for both DEP3 and DEP11), without any difference from each other. In addition, NPY-ir was lower in females than in males (*p* < 0.01). These results are shown in **Figure 6**.

The results of Cohen’s *d* effect size can be found in **Table 4** (comparison between groups, for each sex) and **Table 5** (comparison between sexes, in each group).

**Table 4:** Pairwise calculation of Cohen’s *d* effect size.

Parameter	Males			Females		
	CTL × DEP3	CTL × DEP11	DEP3 × DEP11	CTL × DEP3	CTL × DEP11	DEP3 × DEP11
Body weight	**1.00**	*0.80*	0.4	*0.65*	*0.63*	0.00
Adrenal weight	**1.05**	*0.63*	0.44	**1.57**	**0.85**	0.32
NSF latency	**1.22**	0.18	**0.93**	**1.12**	*0.69*	0.21
NSF intake	0.18	0.46	*0.55*	0.00	0.15	0.17
NSCT Day 3	**1.18**	**1.26**	0.01	0.08	0.23	0.26
NSCT Day 2–Day3	**1.14**	**1.19**	0.11	0.08	0.13	0.39
FST swimming	**4.00**	*0.63*	**2.41**	**2.71**	**1.62**	**1.29**
FST climbing	**0.87**	0.36	**1.22**	**1.73**	*0.69*	**1.21**
FST immobility	**3.69**	*0.59*	**2.61**	**3.08**	**2.01**	**1.63**
% visits to OA	*0.63*	0.22	0.29	**1.42**	0.24	**1.35**
%TOA	**0.96**	0.05	**1.02**	*0.78*	**1.04**	**1.45**
Anxiety index	**1.01**	0.17	**0.86**	**1.16**	*0.53*	**1.44**
Squares in OA	**1.28**	0.07	**1.20**	*0.75*	0.42	**1.13**
Extremity OA	**1.18**	0.12	**1.44**	*0.65*	*0.60*	**1.14**
NPY-ir BLA	**1.89**	**1.41**	0.05	**2.85**	**2.36**	0.33
NPY-ir dHPC	**4.46**	**1.48**	**2.49**	**5.28**	**4.85**	0.09
NPY-ir vHPC	0.09	0.28	0.32	**1.45**	0.34	**0.90**

*Numbers in italic represent moderate magnitude of the effect, whereas bold numbers represent large magnitude of the effect. BLA, basolateral amygdala; dHPC, dorsal hippocampus; CTL, control group; DEP3, rats maternally deprived on postnatal day 3; DEP11, rats maternally deprived on postnatal day 11; FST, forced swimming test; NPY-ir, neuropetide Y immunoreactivity; NSCT, negative sucrose contrast test; NSF, novelty suppressed feeding; OA, open arms; TOA, time in open arms; vHPC, ventral hippocampus.*

**Table 5:** Calculation of Cohen’s *d* effect size between sexes, in each group, for all parameters.

Parameter	Males × Females		
	CTL	DEP3	DEP11
Body weight	**1.60**	**0.81**	**1.61**
Adrenal weight	**2.75**	**1.84**	**2.00**
NSF latency	*0.71*	*0.77*	0.01
NSF intake	**1.34**	**2.20**	**1.10**
NSCT D3	**1.23**	0.31	0.46
NSCT D2–D3	**1.21**	0.18	*0.61*
FST swimming	0.17	*0.71*	*0.56*
FST climbing	**1.04**	0.08	0.07
FST immobility	*0.76*	*0.63*	*0.50*
% visits to OA	**1.15**	0.45	**1.15**
%TOA	0.12	0.20	**1.02**
Anxiety index	*0.72*	0.09	**1.33**
Squares in OA	0.24	0.32	*0.76*
Extremity OA	0.20	0.42	*0.72*
NPY-ir BLA	**0.96**	**1.64**	**0.88**
NPY-ir dHPC	**1.62**	**1.34**	**1.05**
NPY-ir vHPC	*0.57*	**1.36**	0.44

*Numbers in italic represent medium magnitude of the effect, whereas bold numbers represent large magnitude of the effect. BLA, basolateral amygdala; CTL, control group; D2, Day 2; D3, Day 3; DEP3, rats maternally deprived on postnatal day 3; DEP11, rats maternally deprived on postnatal day 11; dHPC, dorsal hippocampus; FST, forced swimming test; NPY-ir, neuropetide Y immunoreactivity; NSCT, negative sucrose contrast test; NSF, novelty suppressed feeding; OA, open arms; TOA, time in open arms; vHPC, ventral hippocampus.*

### Experiment 3

The results of maternal behaviors, including the statistical analysis, are presented in **Table 3**. Of all behaviors recorded, only anogenital licking and grooming was significantly different between the groups. Mothers of DEP11 pups engaged more in this behavior, reaching statistical differences at 17:30 h (*p* < 0.03) and total frequency during the day (*p* < 0.05).

**Table 3:** Frequency (median – 25 and 75 percentile) of the main components of maternal care on the day of reunion of the litters with their mothers.

Behavior/time	Group		Statistics
	DEP3	DEP11	
Passive nursing			
10:00 h	0 (0–0)	0 (0–0)	*U* = 14.0; *p* < 0.7
14:00 h	0 (0–4)	0 (0–0)	*U* = 10.0; *p* < 0.3
17:00 h	0 (0–4)	0 (0–0)	*U* = 15.0; *p* < 0.8
Total	0 (0–5)	0 (0–1)	*U* = 14.0; *p* < 0.7
Blanket nursing			
10:00 h	15 (11–16)	16 (1–17)	*U* = 16.5; *p* < 0.9
14:00 h	16 (14–19)	14 (14–18)	*U* = 15.0; *p* < 0.8
17:00 h	16 (11–20)	11 (10–12)	*U* = 6.5; *p* < 0.08
Total	45 (39–48)	29 (29–42)	*U* = 7.5; *p* < 0.2
Arched-back nursing			
10:00 h	1 (1–2)	2 (0–12)	*U* = 15.0; *p* < 0.8
14:00 h	0 (0–2)	0 (0–1)	*U* = 15.0; *p* < 0.8
17:00 h	0 (0–0)	1 (0–1)	*U* = 11.0; *p* < 0.4
Total	2 (1–6)	2 (2–13)	*U* = 15.0; *p* < 0.8
Licking-grooming			
10:00 h	4 (1–6)	4 (2–5)	*U* = 17.0; *p* = 1.0
14:00 h	1 (1–3)	2 (1–2)	*U* = 16.5; *p* < 0.9
17:00 h	1 (0–2)	0 (0–1)	*U* = 12.5; *p* < 0.5
Total	8 (3–9)	5 (5–7)	*U* = 13.0; *p* < 0.6
Anogenital licking-grooming			
10:00 h	7 (6–9)	9 (7–9)	*U* = 13.5; *p* < 0.6
14:00 h	1 (0–3)	2 (2–3)	*U* = 7.5; *p* < 0.2
17:00 h	0 (0–2)#	5 (2–5)	*U* = 4.0; *p* < 0.03
Total	9 (7–12)#	15 (12–16)	U = 5.0; *p* < 0.05
Interaction with pups			
10:00 h	19 (19–20)	17 (17–19)	*U* = 10.0; *p* < 0.3
14:00 h	20 (16–20)	20 (16–20)	*U* = 17.5; *p* = 1.0
17:00 h	16 (14–20)	16 (16–17)	*U* = 17.0; *p* < 1.0
Total	53 (50–59)	51 (48–53)	*U* = 10.0; *p* < 0.3

*# – Significant difference from DEP11, by the Mann–Whitney U-test (p < 0.05).*

## Discussion

In the present study, we tested the impact of maternal deprivation imposed in two moments of the neonatal development, immediately before (DEP3) and in the midst of the SHRP (DEP11), on physiological variables, such as body weight gain and relative adrenal weight, and on emotional behavior and NPY-ir in the BLA, vHPC, and dHPC of male and female rats. Impairment of body weight gain replicated previous findings (Wertheimer et al., 2016), and in the present study, we also found that females, regardless of their infancy history, showed heavier adrenals than their male counterparts; in addition, maternal deprivation on PND 3 resulted in the heaviest adrenal weights both in males and females, suggesting that DEP3 adolescents may be under chronic stress (Jankord et al., 2011). There was a clear distinctive age-dependent effect of maternal deprivation on emotional behavior, inasmuch as DEP3 adolescents of both sexes displayed the highest anxiety index and the greatest frequency of immobility in the FST; DEP11 adolescents, in turn, displayed more immobility than their CTL counterparts. The greater impact of maternal deprivation at an earlier age recapitulated a recent epidemiological study (Berg et al., 2016), providing an important face validity for this model of vulnerability. Despite this clear behavioral differentiation, the expression of NPY in three well-known areas involved with anxiety- and depressive-like behaviors was altered by maternal deprivation on both periods of development and in both sexes.

All behavioral assessments in females were carried out during the estrous phase, which, together with proestrous, is characterized by lesser anxiety-like profile among the phases of the cycle. For instance, female rats in estrous behave similarly to males in the elevated T maze (Gouveia et al., 2004) and EPM (Marcondes et al., 2001), whereas in the open field, they are less anxious than in the other phases of the cycle (Ramos-Ortolaza et al., 2017). Interestingly, females tested in the EPM and in the punished conflict test are less anxious and more responsive to NPY anxiolytic effect in late proestrous than in metestrous (Molina-Hernández et al., 2006). Comparing all estrous phases in the punished conflict test, it has been observed that during late proestrous and estrous, females are less anxious and more responsive to diazepam than in metestrous and diestrous (Molina-Hernández et al., 2001). Data regarding depressive-like behavior, on the other hand, seem to be controversial; on the one hand, neither the FST (Ramos-Ortolaza et al., 2017) nor the saccharin preference test (Borrow and Cameron, 2017) is influenced by the estrous cycle. On the other hand, findings show that female hormones protect ovariectomized animals in tests of depressive-like behavior (Bredemann and McMahon, 2014; Ibrahim et al., 2016). In the present study, the behaviors displayed by CTL animals in the NSF and EPM were in agreement with the abovementioned findings. The NSF is a test that responds to acute benzodiazepine treatment and to chronic antidepressant administration, being regarded as a test for anxiety-like behavior (Ramaker and Dulawa, 2017), with a strong motivational component. Therefore, we could argue that DEP3 males and females exhibited less motivation to eat in the novel environment. Although the statistical analysis did not detect a difference between CTL and DEP11 females, Cohen’s *d* effect size indicated a moderately relevant effect. On the contrary, contrasting DEP3 and DEP11 females did not reveal any relevance, suggesting that DEP11 females may also display less motivation to eat.

The SNCT test evaluates the response of animals to a shift in reward, by habituating them to a 15% sucrose solution and then shifting it to 2.1%, i.e., a much less palatable solution (Matthews et al., 1996). The behavioral response to change of a familiar solution (15%) with a novel one (2.1%) reflects the comparison between expectancy and reality. Therefore, animals are expected to reduce the intake of the lesser concentrated solution (Matthews et al., 1996). Although all males reduced the 2.1% solution intake, the reduction was greater in the CTL group (approximately 50%, meaning that CTL animals were indifferent to the new solution), whereas DEP3 and DEP11 males still preferred the less palatable solution. The smaller response of deprived animals to the shift in rewarding valence of the stimulus could be interpreted as resembling the blunted hedonic behavior of depressive patients, but this is speculative, for we could not find any published work on the predictive validity of this test. However, the results of the FST are in line with the results of the SNCT, since both manipulated groups, regardless of sex, displayed more immobility and, particularly, DEP3 animals also displayed less climbing than the other groups. The FST is a model of learned helplessness (Porsolt et al., 1977), widely used to assess the efficacy of antidepressant drugs. However, there have been claims that this test assesses the animal’s coping style, providing a distinction between active (climbing and swimming) and passive coping (immobility; de Kloet and Molendijk, 2016); therefore, the FST can be better regarded as a test that objectively detects the depressive-related behavioral state of the animal (Cryan et al., 2005b), since previous exposure to stressors increases immobility time (Weiss et al., 1981). The behaviors displayed in this test can be differentially altered by antidepressants, insofar as noradrenergic-targeted antidepressants increase climbing, whereas serotonin-targeted antidepressants increase swimming time (Cryan et al., 2002, 2005a). The fact that DEP3 animals displayed less climbing and swimming could indicate that the activity of their noradrenergic and serotonergic brain systems was impaired by maternal deprivation. To the best of our knowledge, there have been no reports on the activity of these neurotransmitter systems in DEP3 rats. However, maternal deprivation on PND 9 leads to increased serotonin activity (Rentesi et al., 2010, 2013), whereas on PND 11, maternal deprivation increases 5-HT levels in the hypothalamus and reduces in the frontal cortex (Cabbia et al., 2018), suggesting an age-dependent and region-specific outcome of the neonatal adversity on this neurotransmitter system.

The results of the EPM are in line with reports on differential behavioral response depending on the estrous cycle, at least regarding the spatiotemporal analysis and motor behavior. In addition to these classical parameters, we also assessed the locomotor behavior in the open arms and the frequency of visits to the extremity of the open arms, in order to understand how aversive these sites were to the animals. We observed that DEP3 adolescents also ambulated in and reached the extremity of the open arms less than their CTL and DEP11 counterparts. The profile herein displayed by DEP11 adolescents in anxiety-like behavior tests extends previous findings showing longer exploration of the center part of the open field and less grooming than CTL rats, clearly indicating reduced anxiety-like behavior (Suchecki et al., 2000).

Affective behavior is regulated by a number of neurotransmitters, including noradrenaline (Kalk et al., 2011), serotonin (Ressler and Nemeroff, 2000), and gamma-aminobutyric acid (GABA) (Löw et al., 2000). In the last decades, NPY has gained much attention as a potential anxiolytic, screened in different behavioral tests, such as the Montgomery’s maze, elevated plus maze, and Vogel conflict test (Heilig et al., 1989), having been proposed to both attenuate (Serova et al., 2013) and reverse (Serova et al., 2014) the behavioral alterations induced by a model of PTSD. In addition, pre-clinical and clinical studies indicate NPY involvement with depressive-like behavior and depression, respectively. On the one hand, different classes of antidepressants increase NPY-like immunoreactivity, including tricyclic drugs (Heilig et al., 1988), electroconvulsive therapy (Jiménez-Vasquez et al., 2007), lithium (Husum et al., 2000), and selective serotonin reuptake inhibitors (Nikisch et al., 2005), suggesting that this neuropeptide may be a mediator of several antidepressant therapies. On the other hand, low NPY levels are found in the cerebrospinal fluid of depressed patients (Westrin et al., 1999; Heilig et al., 2004; Ozsoy et al., 2016) and in genetic- (Caberlotto et al., 1998, 1999), lesion- (Goyal et al., 2009), and stress-induced animal models of depression (Sergeyev et al., 2005).

In the present study, maternal deprivation resulted in less NPY-ir in the BLA in both sexes, while an age-dependent differential effect of this early adversity was found in the dHPC of males only, with DEP11 male adolescents presenting a lesser impact than DEP3 ones. With regard to sex differences, levels of NPY-ir were lower in females than in males in all three regions analyzed; females also showed greater vulnerability on tests involving motivational aspects, such as the sucrose preference in the SNCT, as the magnitude of suppression of the less palatable sucrose solution was smaller than that of males, regardless of the infancy history. In fact, evidence indicates that the activity of the NPY system affects motivated behaviors, as demonstrated by studies showing that alcohol-preferring rats display lower NPY levels in the hippocampus, amygdala, and frontal cortex than non-preferring rats (Ehlers et al., 1998), whereas increased production of NPY in the amygdala of transgenic rats prevents escalated ethanol intake in a model of drug dependence (Thorsell et al., 2007).

Several studies evaluating the effects of endogenous NPY show that this neuropeptide is related to stress resilience. For instance, NPY-deficient mice display hypolocomotion and increased anxiety-like behavior measured in the acoustic startle response test (Bannon et al., 2000), open field, elevated plus maze, and light-dark box tests, with males being more affected than females (Karl et al., 2008). In contrast, transgenic rats overexpressing NPY in the CA1 and CA2 areas of the hippocampus are resistant to stress-induced anxiety-like behavior in the elevated plus maze and in the Vogel conflict test (Thorsell et al., 2000). Individual variability in the reaction to a traumatic event leading to PTSD-like behavior was also shown to correlate with NPY levels in the amygdala and periaqueductal gray, i.e., exposed animals showed lower NPY levels than non-exposed and those classified as affected, i.e., exhibiting the greatest behavioral changes, showed lower levels than non-affected animals (Cohen et al., 2012). These findings strongly support the role of NPY as a resilience factor and, based on the expression of this neuropeptide and on the behavioral profile displayed by maternally deprived rats, we hypothesize that maternal deprivation on PNDs 3 and 11 leads not only to increased vulnerability to emotional disorders but most likely to other psychopathological conditions as well, including PTSD and alcohol/drug abuse.

The age-dependent differential effects of maternal deprivation were intriguing; the ages were selected because they represent different stages of the developmental stress response. Thus, at PND 3, pups are more immature, but capable of secreting CORT in response to stressors, whereas at PND 11, pups are more mature but in the midst of the SHRP (Levine et al., 1991). Interestingly, maternal deprivation leads to much higher basal and stress-induced CORT levels in DEP11 than in DEP3 pups (Levine et al., 1991; Suchecki and Tufik, 1997; Faturi et al., 2010). Therefore, we expected DEP11 animals to display more behavioral alterations than DEP3 ones, but the results seemed to point to the opposite direction, suggesting that the impact of maternal deprivation on later behavior and neurobiology may be mediated by factors other than increased CORT levels.

In an attempt to understand the behavioral results of this study, we investigated whether maternal behaviors were different upon reunion with the pups as a function of the pups’ age. This idea was based on the fact that adult animals submitted to neonatal handling, a brief manipulation repeated daily during the pre-weaning period (Levine, 1967) display less fear behavior in the open field and NSF (Caldji et al., 2000b); interestingly, this early life stimulation alters maternal behavior so that mothers increase the frequency of licking and grooming of their pups (Liu et al., 1997). Our results showed that mothers of DEP11 pups engaged more in anogenital licking and grooming than mothers of DEP3 pups, whereas all other behaviors were similar between the groups. This result is remarkable, because the frequency that mothers care for and interact with their pups decreases as a function of the litter’s age (Ader and Grota, 1970; Grota and Ader, 1974). For instance, licking and grooming (Champagne et al., 2003) and nursing bouts (Grota and Ader, 1974) are naturally less frequent in the second than in the first postnatal week and yet, after maternal deprivation no such age difference was observed for nursing and interaction with pups. As for anogenital licking, it is fairly stable from PND 2 until PND 14, in males, whereas it is lower in females than in males from PND 7 on (Moore and Morelli, 1979). Numerous findings demonstrate that the offspring of mothers that display greater frequency of licking and grooming are more resilient to stress and fear-related situations in adulthood (Meaney et al., 1989; Liu et al., 1997; Caldji et al., 2000a; Menard et al., 2004), suggesting that DEP11 animals might be resilient in tests relevant for anxiety behavior because of the level of anogenital licking and grooming received after reunion with the mother. However, a more thorough evaluation of maternal behavior in *Wistar* rats before and after maternal deprivation is worth carrying out in future studies.

## Conclusion

Maternal deprivation resulted in age- and sex-dependent changes in affective behaviors and in the expression of NPY in the basolateral amygdala and dHPC of adolescent rats. Animals deprived on PND 3 were more affected than those deprived on PND11 and females displayed greater changes in motivation-related behaviors than males. Our data suggest that NPY may be a vulnerability factor, but not a main mediator of the effects of maternal deprivation on later behavior, and that increased maternal care toward pups might have conferred greater resilience to DEP11 young adult rats.

## Author Contributions

ASM and GSOW run and collected the data of Experiments 1 and 2. AC and RC run and collected the data of Experiment 3. BL helped with NPY immunohistochemistry, CG participated in the design and data collection of Experiment 2 and writing of the manuscript. DS designed the experiments, helped in the experiments, analyzed the data, and wrote the manuscript. All authors read, edited, and approved the manuscript.

## Conflict of Interest Statement

The authors declare that the research was conducted in the absence of any commercial or financial relationships that could be construed as a potential conflict of interest.

## References

[B1] Ader R. Grota L. J. (1970). Rhythmicity in the maternal behaviour of *Rattus norvegicus*. *Anim. Behav.* 18 144–150. 10.1016/0003-3472(70)90083-7 5530113

[B2] Agid O. Shapira B. Zislin J. Ritsner M. Hanin B. Murad H. (1999). Environment and vulnerability to major psychiatric illness: a case control study of early parental loss in major depression, bipolar disorder and schizophrenia. *Mol. Psychiatry* 4 163–172. 10.1038/sj.mp.4000473 10208448

[B3] Bannon A. W. Seda J. Carmouche M. Francis J. M. Norman M. H. Karbon B. (2000). Behavioral characterization of neuropeptide Y knockout mice. *Brain Res.* 868 79–87. 10.1016/S0006-8993(00)02285-X 10841890

[B4] Barbosa Neto J. B. Tiba P. A. Faturi C. B. de Castro-Neto E. F. da Graça Naffah-Mazacoratti M. de Jesus Mari J. (2012). Stress during development alters anxiety-like behavior and hippocampal neurotransmission in male and female rats. *Neuropharmacology* 62 518–526. 10.1016/j.neuropharm.2011.09.011 21945413

[B5] Baxter A. J. Scott K. M. Vos T. Whiteford H. A. (2013). Global prevalence of anxiety disorders: a systematic review and meta-regression. *Psychol. Med.* 43 897–910. 10.1017/S003329171200147X 22781489

[B6] Bellamy S. Hardy C. (2015). Factors predicting depression across multiple domains in a national longitudinal sample of Canadian youth. *J. Abnorm. Child Psychol.* 43 633–643. 10.1007/s10802-014-9940-3 25240908 PMC4397358

[B7] Berg L. Rostila M. Hjern A. (2016). Parental death during childhood and depression in young adults – A national cohort study. *J. Child Psychol. Psychiatry* 57 1092–1098. 10.1111/jcpp.12560 27058980

[B8] Bodnoff S. R. Suranyi-Cadotte B. Aitken D. H. Quirion R. Meaney M. J. (1988). The effects of chronic antidepressant treatment in an animal model of anxiety. *Psychopharmacology (Berl.)* 95 298–302. 10.1007/BF00181937 3137614

[B9] Borrow A. P. Cameron N. M. (2017). Maternal care and affective behavior in female offspring: implication of the neurosteroid/GABAergic system. *Psychoneuroendocrinology* 76 29–37. 10.1016/j.psyneuen.2016.10.028 27883962

[B10] Bredemann T. M. McMahon L. L. (2014). 17β Estradiol increases resilience and improves hippocampal synaptic function in helpless ovariectomized rats. *Psychoneuroendocrinology* 42 77–88. 10.1016/j.psyneuen.2014.01.004 24636504 PMC4065496

[B11] Cabbia R. Consoli A. Suchecki D. (2018). Association of 24 h maternal deprivation with a saline injection in the neonatal period alters adult stress response and brain monoamines in a sex-dependent fashion. *Stress* [Epub ahead of print]. 10.1080/10253890.2018.1456525 29607713

[B12] Caberlotto L. Fuxe K. Overstreet D. H. Gerrard P. Hurd Y. L. (1998). Alterations in neuropeptide Y and Y1 receptor mRNA expression in brains from an animal model of depression: region specific adaptation after fluoxetine treatment. *Brain Res. Mol. Brain Res.* 59 58–65. 10.1016/S0169-328X(98)00137-5 9729278

[B13] Caberlotto L. Jimenez P. Overstreet D. H. Hurd Y. L. Mathé A. A. Fuxe K. (1999). Alterations in neuropeptide Y levels and Y1 binding sites in the flinders sensitive line rats, a genetic animal model of depression. *Neurosci. Lett.* 265 191–194. 10.1016/S0304-3940(99)00234-7 10327163

[B14] Caldji C. Diorio J. Meaney M. J. (2000a). Variations in maternal care in infancy regulate the development of stress reactivity. *Biol. Psychiatry* 48 1164–1174. 10.1016/S0006-3223(00)01084-2 11137058

[B15] Caldji C. Francis D. Sharma S. Plotsky P. M. Meaney M. J. (2000b). The effects of early rearing environment on the development of GABAA and central benzodiazepine receptor levels and novelty-induced fearfulness in the rat. *Neuropsychopharmacology* 22 219–229. 10.1016/S0893-133X(99)00110-4 10693149

[B16] Caldji C. Tannenbaum B. Sharma S. Francis D. Plotsky P. M. Meaney M. J. (1998). Maternal care during infancy regulates the development of neural systems mediating the expression of fearfulness in the rat. *Proc. Natl. Acad. Sci. U.S.A.* 95 5335–5340. 10.1073/pnas.95.9.5335 9560276 PMC20261

[B17] Caspi A. Sugden K. Moffitt T. E. Taylor A. Craig I. W. Harrington H. (2003). Influence of life stress on depression: moderation by a polymorphism in the 5-HTT gene. *Science* 301 386–389. 10.1126/science.1083968 12869766

[B18] Champagne F. A. Francis D. D. Mar A. Meaney M. J. (2003). Variations in maternal care in the rat as a mediating influence for the effects of environment on development. *Physiol. Behav.* 79 359–371. 10.1016/S0031-9384(03)00149-5 12954431

[B19] Cirulli F. Gottlieb S. L. Rosenfeld P. Levine S. (1992). Maternal factors regulate stress responsiveness in the neonatal rat. *Psychobiology* 20 143–152.

[B20] Cohen H. Liu T. Kozlovsky N. Kaplan Z. Zohar J. Mathé A. A. (2012). The neuropeptide Y (NPY)-ergic system is associated with behavioral resilience to stress exposure in an animal model of post-traumatic stress disorder. *Neuropsychopharmacology* 37 350–363. 10.1038/npp.2011.230 21976046 PMC3242318

[B21] Cohen H. Zohar J. Matar M. (2003). The relevance of differential response to trauma in an animal model of posttraumatic stress disorder. *Biol. Psychiatry* 53 463–473. 10.1016/S0006-3223(02)01909-1 12644351

[B22] Cryan J. F. Page M. E. Lucki I. (2002). Noradrenergic lesions differentially alter the antidepressant-like effects of reboxetine in a modified forced swim test. *Eur. J. Pharmacol.* 436 197–205. 10.1016/S0014-2999(01)01628-4 11858799

[B23] Cryan J. F. Page M. E. Lucki I. (2005a). Differential behavioral effects of the antidepressants reboxetine, fluoxetine, and moclobemide in a modified forced swim test following chronic treatment. *Psychopharmacology (Berl.)* 182 335–344. 10.1007/s00213-005-0093-5 16001105

[B24] Cryan J. F. Valentino R. J. Lucki I. (2005b). Assessing substrates underlying the behavioral effects of antidepressants using the modified rat forced swimming test. *Neurosci. Biobehav. Rev.* 29 547–569. 10.1016/j.neubiorev.2005.03.008 15893822

[B25] de Kloet E. R. Molendijk M. L. (2016). Coping with the forced swim stressor: towards understanding an adaptive mechanism. *Neural Plast.* 2016:6503162. 10.1155/2016/6503162 27034848 PMC4806646

[B26] Detke M. J. Rickels M. Lucki I. (1995). Active behaviors in the rat forced swimming test differentially produced by serotonergic and noradrenergic antidepressants. *Psychopharmacology (Berl.)* 121 66–72. 10.1007/BF02245592 8539342

[B27] Ehlers C. L. Li T. K. Lumeng L. Hwang B. H. Somes C. Jimenez P. (1998). Neuropeptide Y levels in ethanol-naive alcohol-preferring and nonpreferring rats and in Wistar rats after ethanol exposure. *Alcohol. Clin. Exp. Res.* 22 1778–1782. 10.1111/j.1530-0277.1998.tb03979.x 9835294

[B28] Ellenbroek B. A. van den Kroonenberg P. T. Cools A. R. (1998). The effects of an early stressful life event on sensorimotor gating in adult rats. *Schizophr. Res.* 30 251–260. 10.1016/S0920-9964(97)00149-7 9589519

[B29] Faturi C. B. Tiba P. A. Kawakami S. E. Catallani B. Kerstens M. Suchecki D. (2010). Disruptions of the mother-infant relationship and stress-related behaviours: altered corticosterone secretion does not explain everything. *Neurosci. Biobehav. Rev.* 34 821–834. 10.1016/j.neubiorev.2009.09.002 19751762

[B30] Girardi C. E. Zanta N. C. Suchecki D. (2014). Neonatal stress-induced affective changes in adolescent Wistar rats: early signs of schizophrenia-like behavior. *Front. Behav. Neurosci.* 8:319. 10.3389/fnbeh.2014.00319 25309370 PMC4159973

[B31] Gouveia A. dos Santos U. D. Felisbino F. E. de Afonseca T. L. Antunes G. Morato S. (2004). Influence of the estrous cycle on the behavior of rats in the elevated T-maze. *Behav. Processes.* 67 167–171. 10.1016/j.beproc.2004.03.018 15240054

[B32] Goyal S. N. Upadhya M. A. Kokare D. M. Bhisikar S. M. Subhedar N. K. (2009). Neuropeptide Y modulates the antidepressant activity of imipramine in olfactory bulbectomized rats: involvement of NPY Y1 receptors. *Brain Res.* 1266 45–53. 10.1016/j.brainres.2009.02.033 19254701

[B33] Grota L. J. Ader R. (1974). Behavior of lactating rats in a dual-chambered maternity cage. *Horm. Behav.* 5 275–282. 10.1016/0018-506X(74)90014-2 4477150

[B34] Gunnar M. R. Cheatham C. L. (2003). Brain and behavior interface: stress and the developing brain. *Infant Mental Health J.* 24 195 –211. 10.1002/imhj.10052

[B35] Heilig M. (2004). The NPY system in stress, anxiety and depression. *Neuropeptides* 38 213–224. 10.1016/j.npep.2004.05.002 15337373

[B36] Heilig M. Söderpalm B. Engel J. A. Widerlöv E. (1989). Centrally administered neuropeptide Y (NPY) produces anxiolytic-like effects in animal anxiety models. *Psychopharmacology (Berl.)* 98 524–529. 10.1007/BF00441953 2570434

[B37] Heilig M. Wahlestedt C. Ekman R. Widerlöv E. (1988). Antidepressant drugs increase the concentration of neuropeptide Y (NPY)-like immunoreactivity in the rat brain. *Eur. J. Pharmacol.* 147 465–467. 10.1016/0014-2999(88)90182-3 2967771

[B38] Heilig M. Zachrisson O. Thorsell A. Ehnvall A. Mottagui-Tabar S. Sjögren M. (2004). Decreased cerebrospinal fluid neuropeptide Y (NPY) in patients with treatment refractory unipolar major depression: preliminary evidence for association with preproNPY gene polymorphism. *J. Psychiatr. Res.* 38 113–121. 10.1016/S0022-3956(03)00101-8 14757324

[B39] Hodel A. S. Hunt R. H. Cowell R. A. Van Den Heuvel S. E. Gunnar M. R. Thomas K. M. (2015). Duration of early adversity and structural brain development in post-institutionalized adolescents. *Neuroimage* 105 112–119. 10.1016/j.neuroimage.2014.10.020 25451478 PMC4262668

[B40] Hofer M. A. (1994). Hidden regulators in attachment, separation, and loss. *Monogr. Soc. Res. Child Dev.* 59 192–207. 10.2307/1166146 7984161

[B41] Huot R. L. Thrivikraman K. V. Meaney M. J. Plotsky P. M. (2001). Development of adult ethanol preference and anxiety as a consequence of neonatal maternal separation in Long Evans rats and reversal with antidepressant treatment. *Psychopharmacology (Berl.)* 158 366–373. 10.1007/s002130100701 11797057

[B42] Husum H. Mathé A. A. (2002). Early life stress changes concentrations of neuropeptide Y and corticotropin-releasing hormone in adult rat brain. Lithium treatment modifies these changes. *Neuropsychopharmacology* 27 756–764. 10.1016/S0893-133X(02)00363-9 12431850

[B43] Husum H. Mikkelsen J. D. Hogg S. Mathé A. A. Mørk A. (2000). Involvement of hippocampal neuropeptide Y in mediating the chronic actions of lithium, electroconvulsive stimulation and citalopram. *Neuropharmacology* 39 1463–1473. 10.1016/S0028-3908(00)00009-5 10818262

[B44] Husum H. Termeer E. Mathé A. A. Bolwig T. G. Ellenbroek B. A. (2002). Early maternal deprivation alters hippocampal levels of neuropeptide Y and calcitonin-gene related peptide in adult rats. *Neuropharmacology* 42 798–806. 10.1016/S0028-3908(02)00038-2 12015206

[B45] Ibrahim W. W. Safar M. M. Khattab M. M. Agha A. M. (2016). 17β-Estradiol augments antidepressant efficacy of escitalopram in ovariectomized rats: neuroprotective and serotonin reuptake transporter modulatory effects. *Psychoneuroendocrinology* 74 240–250. 10.1016/j.psyneuen.2016.09.013 27685339

[B46] Jankord R. Solomon M. B. Albertz J. Flak J. N. Zhang R. Herman J. P. (2011). Stress vulnerability during adolescent development in rats. *Endocrinology* 152 629–638. 10.1210/en.2010-0658 21106877 PMC3037163

[B47] Jiménez-Vasquez P. A. Diaz-Cabiale Z. Caberlotto L. Bellido I. Overstreet D. Fuxe K. (2007). Electroconvulsive stimuli selectively affect behavior and neuropeptide Y (NPY) and NPY Y(1) receptor gene expressions in hippocampus and hypothalamus of Flinders Sensitive Line rat model of depression. *Eur. Neuropsychopharmacol.* 17 298–308. 10.1016/j.euroneuro.2006.06.011 16904299

[B48] Jiménez-Vasquez P. A. Mathé A. A. Thomas J. D. Riley E. P. Ehlers C. L. (2001). Early maternal separation alters neuropeptide Y concentrations in selected brain regions in adult rats. *Brain Res. Dev. Brain Res.* 131 149–152. 10.1016/S0165-3806(01)00264-4 11718845

[B49] Kalk N. J. Nutt D. J. Lingford-Hughes A. R. (2011). The role of central noradrenergic dysregulation in anxiety disorders: evidence from clinical studies. *J. Psychopharmacol.* 25 3–16. 10.1177/0269881110367448 20530586

[B50] Karl T. Duffy L. Herzog H. (2008). Behavioural profile of a new mouse model for NPY deficiency. *Eur. J. Neurosci.* 28 173–180. 10.1111/j.1460-9568.2008.06306.x 18616565

[B51] Kormos V. Gaszner B. (2013). Role of neuropeptides in anxiety, stress, and depression: from animals to humans. *Neuropeptides* 47 401–419. 10.1016/j.npep.2013.10.014 24210138

[B52] Levine S. (1967). Maternal and environmental influences on the adrenocortical response to stress in weanling rats. *Science* 156 258–260. 10.1126/science.156.3772.258 6021047

[B53] Levine S. Huchton D. M. Wiener S. G. Rosenfeld P. (1991). Time course of the effect of maternal deprivation on the hypothalamic-pituitary-adrenal axis in the infant rat. *Dev. Psychobiol.* 24 547–558. 10.1002/dev.420240803 1773913

[B54] Liu D. Diorio J. Tannenbaum B. Caldji C. Francis D. Freedman A. (1997). Maternal care, hippocampal glucocorticoid receptors, and hypothalamic-pituitary-adrenal responses to stress. *Science* 277 1659–1662. 10.1126/science.277.5332.1659 9287218

[B55] Loi M. Koricka S. Lucassen P. J. Joëls M. (2014). Age- and sex-dependent effects of early life stress on hippocampal neurogenesis. *Front. Endocrinol. (Lausanne)* 5:13 10.3389/fendo.2014.00013 PMC3929839 24600436

[B56] Loman M. M. Johnson A. E. Quevedo K. Lafavor T. L. Gunnar M. R. (2014). Risk-taking and sensation-seeking propensity in postinstitutionalized early adolescents. *J. Child Psychol. Psychiatry* 55 1145–1152. 10.1111/jcpp.12208 24552550 PMC4138294

[B57] Löw K. Crestani F. Keist R. Benke D. Brünig I. Benson J. A. (2000). Molecular and neuronal substrate for the selective attenuation of anxiety. *Science* 290 131–134. 10.1126/science.290.5489.131 11021797

[B58] Lucki I. (1997). The forced swimming test as a model for core and component behavioral effects of antidepressant drugs. *Behav. Pharmacol.* 8 523–532. 10.1097/00008877-199711000-00010 9832966

[B59] Marcondes F. K. Miguel K. J. Melo L. L. Spadari-Bratfisch R. C. (2001). Estrous cycle influences the response of female rats in the elevated plus-maze test. *Physiol. Behav.* 74 435–440. 10.1016/S0031-9384(01)00593-5 11790402

[B60] Matthews K. Wilkinson L. S. Robbins T. W. (1996). Repeated maternal separation of preweanling rats attenuates behavioral responses to primary and conditioned incentives in adulthood. *Physiol. Behav.* 59 99–107. 10.1016/0031-9384(95)02069-1 8848498

[B61] Mazor A. Matar M. A. Kaplan Z. Kozlovsky N. Zohar J. Cohen H. (2009). Gender-related qualitative differences in baseline and post-stress anxiety responses are not reflected in the incidence of criterion-based PTSD-like behaviour patterns. *World J Biol Psychiatry* 10 856–869. 10.1080/15622970701561383 17886167

[B62] Meaney M. J. Aitken D. H. Viau V. Sharma S. Sarrieau A. (1989). Neonatal handling alters adrenocortical negative feedback sensitivity and hippocampal type II glucocorticoid receptor binding in the rat. *Neuroendocrinology* 50 597–604. 10.1159/000125287 2558328

[B63] Menard J. L. Champagne D. L. Meaney M. J. (2004). Variations of maternal care differentially influence ‘fear’ reactivity and regional patterns of cFos immunoreactivity in response to the shock-probe burying test. *Neuroscience* 129 297–308. 10.1016/j.neuroscience.2004.08.009 15501588

[B64] Molina-Hernández M. Contreras C. M. Téllez-Alcántara P. (2001). Diazepam increases the number of punished responses in a conflict-operant paradigm during late proestrus and estrus in the Wistar rat. *Neuropsychobiology* 43 29–33. 10.1159/000054862 11150896

[B65] Molina-Hernández M. Olivera-Lopez J. I. Patricia Tellez-Alcántara N. Pérez-García J. Teresa Jaramillo M. (2006). Estrus variation in anxiolytic-like effects of intra-lateral septal infusions of the neuropeptide Y in Wistar rats in two animal models of anxiety-like behavior. *Peptides* 27 2722–2730. 10.1016/j.peptides.2006.05.017 16806581

[B66] Moore C. L. Morelli G. A. (1979). Mother rats interact differently with male and female offspring. *J. Comp. Physiol. Psychol.* 93 677–684. 10.1037/h0077599 479402

[B67] Nikisch G. Agren H. Eap C. B. Czernik A. Baumann P. Mathé A. A. (2005). Neuropeptide Y and corticotropin-releasing hormone in CSF mark response to antidepressive treatment with citalopram. *Int. J. Neuropsychopharmacol.* 8 403–410. 10.1017/S1461145705005158 15784158

[B68] Oomen C. A. Girardi C. E. Cahyadi R. Verbeek E. C. Krugers H. Joëls M. (2009). Opposite effects of early maternal deprivation on neurogenesis in male versus female rats. *PLoS One* 4:e3675. 10.1371/journal.pone.0003675 19180242 PMC2629844

[B69] Oomen C. A. Soeters H. Audureau N. Vermunt L. van Hasselt F. N. Manders E. M. (2010). Severe early life stress hampers spatial learning and neurogenesis, but improves hippocampal synaptic plasticity and emotional learning under high-stress conditions in adulthood. *J. Neurosci.* 30 6635–6645. 10.1523/JNEUROSCI.0247-10.2010 20463226 PMC6632559

[B70] Ozsoy S. Olguner Eker O. Abdulrezzak U. (2016). The effects of antidepressants on neuropeptide Y in patients with depression and anxiety. *Pharmacopsychiatry* 49 26–31. 10.1055/s-0035-1565241 26789271

[B71] Patten S. B. Wang J. L. Williams J. V. Currie S. Beck C. A. Maxwell C. J. (2006). Descriptive epidemiology of major depression in Canada. *Can. J. Psychiatry* 51 84–90. 10.1177/070674370605100204 16989107

[B72] Paxinos G. Watson C. (1998). *The Rat Brain in Stereotaxic Coordinates*, 4th Edn. San Diego, CA: Academic Press, 237.

[B73] Pellow S. Chopin P. File S. E. Briley M. (1985). Validation of open:closed arm entries in an elevated plus-maze as a measure of anxiety in the rat. *J. Neurosci. Methods* 14 149–167. 10.1016/0165-0270(85)90031-7 2864480

[B74] Plotsky P. M. Owens M. J. Nemeroff C. B. (1998). Psychoneuroendocrinology of depression. Hypothalamic-pituitary-adrenal axis. *Psychiatr. Clin. N. Am.* 21 293–307. 10.1016/S0193-953X(05)70006-X 9670227

[B75] Porsolt R. D. Bertin A. Jalfre M. (1977). Behavioral despair in mice: a primary screening test for antidepressants. *Arch Int. Pharmacodyn. Ther.* 229 327–336. 596982

[B76] Ramaker M. J. Dulawa S. C. (2017). Identifying fast-onset antidepressants using rodent models. *Mol. Psychiatry* 22 656–665. 10.1038/mp.2017.36 28322276

[B77] Ramos-Ortolaza D. L. Doreste-Mendez R. J. Alvarado-Torres J. K. Torres-Reveron A. (2017). Ovarian hormones modify anxiety behavior and glucocorticoid receptors after chronic social isolation stress. *Behav. Brain Res.* 328 115–122. 10.1016/j.bbr.2017.04.016 28408299 PMC5772780

[B78] Rees S. L. Lovic V. Fleming A. S. (2005). “Maternal behavior,” in *The Behavior of the Laboratory Rat: A Handbook With Tests*, eds Whishaw I. Q. Kolb B. (New York, NY: Oxford Scholarship), 304–314.

[B79] Rentesi G. Antoniou K. Marselos M. Fotopoulos A. Alboycharali J. Konstandi M. (2010). Long-term consequences of early maternal deprivation in serotonergic activity and HPA function in adult rat. *Neurosci. Lett.* 480 7–11. 10.1016/j.neulet.2010.04.054 20435091

[B80] Rentesi G. Antoniou K. Marselos M. Syrrou M. Papadopoulou-Daifoti Z. Konstandi M. (2013). Early maternal deprivation-induced modifications in the neurobiological, neurochemical and behavioral profile of adult rats. *Behav. Brain Res.* 244 29–37. 10.1016/j.bbr.2013.01.040 23395600

[B81] Ressler K. J. Nemeroff C. B. (2000). Role of serotonergic and noradrenergic systems in the pathophysiology of depression and anxiety disorders. *Depress. Anxiety* 12 Suppl 1 2–19. 10.1002/1520-6394(2000)12:1+<2::AID-DA2>3.0.CO;2-4 11098410

[B82] Rosenfeld P. Ekstrand J. Olson E. Suchecki D. Levine S. (1993). Maternal regulation of adrenocortical activity in the infant rat: effects of feeding. *Dev. Psychobiol.* 26 261–277. 10.1002/dev.420260504 8339865

[B83] Rosenfeld P. Suchecki D. Levine S. (1992). Multifactorial regulation of the hypothalamic-pituitary-adrenal axis during development. *Neurosci. Biobehav. Rev.* 16 553–568. 10.1016/S0149-7634(05)80196-4 1480351

[B84] Sah R. Ekhator N. N. Jefferson-Wilson L. Horn P. S. Geracioti T. D. (2014). Cerebrospinal fluid neuropeptide Y in combat veterans with and without posttraumatic stress disorder. *Psychoneuroendocrinology* 40 277–283. 10.1016/j.psyneuen.2013.10.017 24485499 PMC4749916

[B85] Sah R. Ekhator N. N. Strawn J. R. Sallee F. R. Baker D. G. Horn P. S. (2009). Low cerebrospinal fluid neuropeptide Y concentrations in posttraumatic stress disorder. *Biol. Psychiatry* 66 705–707. 10.1016/j.biopsych.2009.04.037 19576571 PMC4751867

[B86] Sergeyev V. Fetissov S. Mathé A. A. Jimenez P. A. Bartfai T. Mortas P. (2005). Neuropeptide expression in rats exposed to chronic mild stresses. *Psychopharmacology (Berl.)* 178 115–124. 10.1007/s00213-004-2015-3 15719227

[B87] Serova L. I. Laukova M. Alaluf L. G. Pucillo L. Sabban E. L. (2014). Intranasal neuropeptide Y reverses anxiety and depressive-like behavior impaired by single prolonged stress PTSD model. *Eur. Neuropsychopharmacol.* 24 142–147. 10.1016/j.euroneuro.2013.11.007 24326087

[B88] Serova L. I. Tillinger A. Alaluf L. G. Laukova M. Keegan K. Sabban E. L. (2013). Single intranasal neuropeptide Y infusion attenuates development of PTSD-like symptoms to traumatic stress in rats. *Neuroscience* 236 298–312. 10.1016/j.neuroscience.2013.01.040 23376740

[B89] Suchecki D. Tufik S. (1997). Long-term effects of maternal deprivation on the corticosterone response to stress in rats. *Am. J. Physiol. Regul. Integr. Compar. Physiol.* 273 R1332–R1338. 10.1152/ajpregu.1997.273.4.R1332 9362296

[B90] Suchecki D. Duarte Palma B. Tufik S. (2000). Pituitary-adrenal axis and behavioural responses of maternally deprived juvenile rats to the open field. *Behav. Brain Res.* 111 99–106. 10.1016/S0166-4328(00)00148-0 10840136

[B91] Suchecki D. Rosenfeld P. Levine S. (1993). Maternal regulation of the hypothalamic-pituitary-adrenal axis in the infant rat: the roles of feeding and stroking. *Brain Res. Dev. Brain Res.* 75 185–192. 10.1016/0165-3806(93)90022-3 8261610

[B92] ter Horst J. P. de Kloet E. R. Schächinger H. Oitzl M. S. (2012). Relevance of stress and female sex hormones for emotion and cognition. *Cell Mol. Neurobiol* 32 725–735. 10.1007/s10571-011-9774-2 22113371 PMC3377901

[B93] Thorsell A. Mathé A. A. (2017). Neuropeptide Y in alcohol addiction and affective disorders. *Front. Endocrinol. (Lausanne)* 8:178 10.3389/fendo.2017.00178 PMC5534438 28824541

[B94] Thorsell A. Michalkiewicz M. Dumont Y. Quirion R. Caberlotto L. Rimondini R. (2000). Behavioral insensitivity to restraint stress, absent fear suppression of behavior and impaired spatial learning in transgenic rats with hippocampal neuropeptide Y overexpression. *Proc. Natl. Acad. Sci. U.S.A.* 97 12852–12857. 10.1073/pnas.220232997 11058155 PMC18853

[B95] Thorsell A. Repunte-Canonigo V. O’Dell L. E. Chen S. A. King A. R. Lekic D. (2007). Viral vector-induced amygdala NPY overexpression reverses increased alcohol intake caused by repeated deprivations in Wistar rats. *Brain* 130 1330–1337. 10.1093/brain/awm033 17405766 PMC2749684

[B96] Tofoli S. M. C. Von Werne Baes C. Martins C. M. S. Juruena M. (2011). Early life stress, HPA axis, and depression. *Psychol.Neurosci.* 4 229–234. 10.3922/j.psns.2011.2.008

[B97] Tottenham N. Hare T. A. Quinn B. T. McCarry T. W. Nurse M. Gilhooly T. (2010). Prolonged institutional rearing is associated with atypically large amygdala volume and difficulties in emotion regulation. *Dev. Sci.* 13 46–61. 10.1111/j.1467-7687.2009.00852.x 20121862 PMC2817950

[B98] Tyrka A. R. Wier L. Price L. H. Ross N. Anderson G. M. Wilkinson C. W. (2008). Childhood parental loss and adult hypothalamic-pituitary-adrenal function. *Biol. Psychiatry* 63 1147–1154. 10.1016/j.biopsych.2008.01.011 18339361 PMC2650434

[B99] van Oers H. J. de Kloet E. R. Whelan T. Levine S. (1998). Maternal deprivation effect on the infant’s neural stress markers is reversed by tactile stimulation and feeding but not by suppressing corticosterone. *J. Neurosci.* 18 10171–10179. 10.1523/JNEUROSCI.18-23-10171.1998 9822770 PMC6793306

[B100] Verma P. Hellemans K. G. Choi F. Y. Yu W. Weinberg J. (2010). Circadian phase and sex effects on depressive/anxiety-like behaviors and HPA axis responses to acute stress. *Physiol. Behav.* 99 276–285. 10.1016/j.physbeh.2009.11.002 19932127 PMC2856664

[B101] Walf A. A. Frye C. A. (2010). Raloxifene and/or estradiol decrease anxiety-like and depressive-like behavior, whereas only estradiol increases carcinogen-induced tumorigenesis and uterine proliferation among ovariectomized rats. *Behav. Pharmacol.* 21 231–240. 10.1097/FBP.0b013e32833a5cb0 20480545 PMC2885355

[B102] Weiss J. M. Goodman P. A. Losito B. G. Corrigan S. Charry J. M. Bailey W. H. (1981). Behavioral depression produced by an uncontrollable stressor: relationship to norepinephrine, dopamine, and serotonin levels in various regions of rat brain. *Brain Res. Rev.* 3 167–205. 10.1016/0165-0173(81)90005-9

[B103] Wertheimer G. S. Girardi C. E. de Oliveira A. S. Monteiro Longo B. Suchecki D. (2016). Maternal deprivation alters growth, food intake, and neuropeptide Y in the hypothalamus of adolescent male and female rats. *Dev. Psychobiol.* 58 1066–1075. 10.1002/dev.21440 27307308

[B104] Westrin A. Ekman R. Träskman-Bendz L. (1999). Alterations of corticotropin releasing hormone (CRH) and neuropeptide Y (NPY) plasma levels in mood disorder patients with a recent suicide attempt. *Eur. Neuropsychopharmacol.* 9 205–211. 10.1016/S0924-977X(98)00026-1 10208289

[B105] WHO (2018). *World Health Organization – Depression.* Available atL http://www.who.int/en/news-room/fact-sheets/detail/depression

[B106] Witek-Janusek L. (1988). Pituitary-adrenal response to bacterial endotoxin in developing rats. *Am. J. Physiol.* 255 E525–E530. 10.1152/ajpendo.1988.255.4.E525 2845803

[B107] Wu G. Feder A. Wegener G. Bailey C. Saxena S. Charney D. (2011). Central functions of neuropeptide Y in mood and anxiety disorders. *Exp. Opin. Ther. Targets* 15 1317–1331. 10.1517/14728222.2011.628314 21995655

